# Synthesis of size-controlled PtPdIr nanoparticles by solution plasma sputtering and their catalytic properties†

**DOI:** 10.1039/d5ra01747e

**Published:** 2025-04-22

**Authors:** Yuanyuan Liu, Zhunda Zhu, Zhuoya Deng, Pengfei Wang, Sangwoo Chae, Yasuyuki Sawada, Nagahiro Saito

**Affiliations:** a Department of Chemical Systems Engineering, Graduate School of Engineering, Nagoya University Furo-cho, Chikusa-ku Nagoya 464-8603 Japan saito.nagahiro.z7@f.mail.nagoya-u.ac.jp; b Institute of Innovation for Future Society, Nagoya University Furo-cho, Chikusa-ku Nagoya 464-8603 Japan; c Department of International Collaborative Program in Sustainable Materials and Technology for Industries between Nagoya University and Chulalongkorn University, Graduate School of Engineering, Nagoya University Furo-cho, Chikusa-ku Nagoya 464-8603 Japan; d Conjoint Research Laboratory in Nagoya University, Shinshu University Furo-cho, Chikusa-ku Nagoya 464-8603 Japan

## Abstract

Platinum-based catalysts are widely used in polymer electrolyte fuel cells (PEMFCs) due to their excellent catalytic activity for the oxygen reduction reaction (ORR) and hydrogen oxidation reaction (HOR). In this study, a PtPdIr ternary alloy catalyst was synthesized by a solution plasma (SP) sputtering process with PtPd and PtIr erelctrodes, which provides a non-equilibrium reaction field in solution. The ratio of Ir in the PtPdIr nanoparticles increased as the ratio of Ir in the PtIr electrode increased. However, the ratio reamined constant at about 10%. The size of the nanoparticles could be controlled in the range of 1–3 nm. In addition, the nanoparticles were well dispersed when supported on carbon and no agglomeration was observed. The electrochemical properties of the obtained nanoparticles were investigated in terms of ORR and HOR, and the particle-c (79 : 14 : 7) nanoparticle exhibited the highest ORR and HOR performance. XPS analysis showed that the intensity of I_Pd(ii)_ and I_Pd(0)_ in particle-c (79 : 14 : 7) was at the same level, and that the chemical bonding state of these elements enhances ORR and HOR activity.

## Abstract 2

PtPdIr ternary alloys with controlled composition and size synthesized *via* solution plasma sputtering process for oxygen reduction reaction and hydrogen oxidation reaction catalysis.

## Graphical abstract

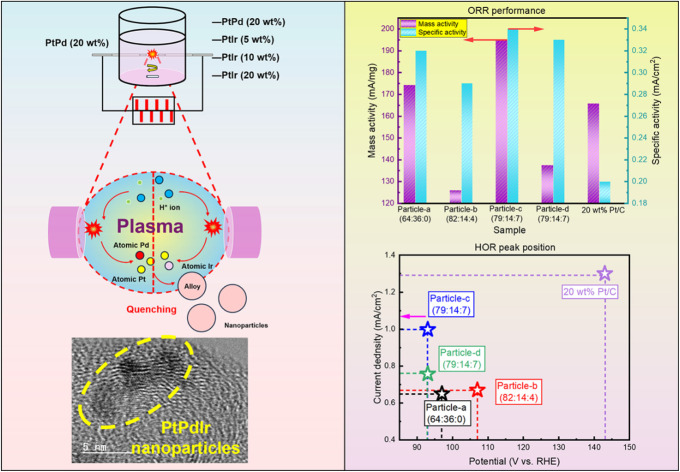

## 1. Introduction

Carbon dioxide emissions have increased significantly since the 1850s and were projected to reach approximately 50 billion tons worldwide by 2020. Fossil fuel combustion accounts for more than two-thirds of total emissions.^1,2^ Transportation, which is essential to modern society, is a major contributor to these emissions. Compared to conventional internal combustion engine vehicles (ICEVs), electric vehicles (EVs) have many advantages, including high energy efficiency, zero carbon dioxide emissions, and significant reductions in operating costs and electricity bills. These characteristics make fuel cell and lithium-ion battery electric vehicles a cleaner, and more sustainable alternative to conventional vehicles. Direct methanol fuel cells, which use biomass ethanol and air as fuel, do emit CO_2_, but because it is carbon derived from natural sources, the effect of reducing CO_2_ emissions is believed to be significant.^3,4^ The advantages of using direct ethanol fuel cells include the ease of handling due to ethanol being a liquid fuel, the system's simplicity resulting from the use of direct ethanol, and the high energy density attributed to its liquid state. On the other hand, the power density is low, methanol crossover occurs, and the catalyst is poisoned.^5^ The development of direct ethanol fuel cells is limited by the high cost of catalysts and issues related to catalyst stability and durability, which are the main obstacles to large-scale commercialization.^6^ Platinum (Pt) is recognized as the most effective catalytic metal and is widely used in fuel cell electrodes.^7–9^ However, the slow rate of the oxygen reduction reaction (ORR) at the cathode and the high cost of platinum-based catalysts are common problems with fuel cell catalysts, and these factors significantly hinder the widespread adoption of fuel cells.^10,11^ In addition, direct ethanol fuel cells are still plagued by catalyst poisoning^12^ and the slow oxidation of ethanol compared to hydrogen.^13^ Therefore, reducing catalyst cost, improving performance, and increasing durability have become important priorities in direct ethanol fuel cell research.^14,15^

A common approach to improving catalyst performance is the development of platinum-based alloys. These alloys allow charge transfer between Pt and other metals and can enhance catalytic activity by effectively tuning the electronic structure, particularly the d-band center.^16–20^ Among the candidate alloying elements, palladium (Pd) has attracted much attention because it is relatively abundant and inexpensive compared to platinum (Pt), and it has the ability to tune the electronic structure of Pt while maintaining excellent catalytic performance. For example, Zhang *et al.*^21^ used a solution plasma (SP) sputtering process to synthesize a PtPd binary alloy for use in methanol fuel cells, achieving four times the electrocatalytic activity of commercial Pt/C catalysts. Similarly, Rivera-Lugo *et al.*^22^ reported that they successfully achieved direct reduction of PtPd catalysts on rGO and SWCNTs, resulting in ORR catalysts with excellent performance and stability. In addition, Duan and his team demonstrated that the 12.3 nm PtPd alloy catalysts obtained by dealloying treatment showed excellent ORR activity, and the NP-Pt75Pd25 alloy achieved the highest catalytic performance.^23^

Iridium (Ir), which is the second element after platinum in the periodic table, is known to exhibit excellent catalytic performance in the oxygen evolution reaction (OER).^24–26^ Pt and Ir have the same crystal structure and similar lattice constants, so the lattice mismatch is minimized. In addition, because the standard electrode potential of Ir is high, the stability of Pt is greatly improved when they are alloyed. Because of these advantages, Ir-containing PtIr alloys are very promising for use as an ORR catalyst. Kusunoki *et al.*^16^ synthesized Ir-modified PtPd alloys and found that charge transfer between Pt and Ir can enhance ORR activity. Kai Deng *et al.*^17^ prepared bifunctional mesoporous hollow PtPdIr nanospheres, which exhibited catalytic activity for both methanol oxidation reaction (MOR) and ORR. Bifunctional mesoporous hollow PtPdIr nanospheres were prepared. Zhu *et al.*^27^ synthesized cubic Pt39Ir10Pd11 nanocages and showed high mass activity of 0.52 A mg^−1^ Pt + Ir + Pd. This was about twice the value of Pt/C. From previous results, it can be said that it is difficult to control the shape of PtPdIr particles, although in general, spherical particles with a diameter of 1–3 nm are often good in terms of catalytic activity and stability.^17,28^

In recent years, research has become more active in the field of multi-element alloys, such as high-entropy and medium-entropy alloys, with the aim of further adjusting the d-band center and providing multiple functions by coexisting with other elements.^29–31^ However, in the case of multi-element nanoparticles, the difficulty of controlling the size, shape, composition, *etc.* also increases. The methods commonly used to synthesize metal nanoparticles include chemical reduction,^30^ hydrothermal synthesis,^32^ and etching.^27^ These methods have low reaction efficiency, require high temperatures and pressures, reducing agents and reducing gases, and the experimental process is complex and time-consuming. Strong interactions between thermodynamic equilibrium reactions characterize these reaction processes.

There has been progress in the technology of nanoparticle synthesis using the solution plasma process (SPP).^33–35^ SPP is a one-step method for the synthesis of metal nanoparticles at room temperature and atmospheric pressure without the use of reducing agents. In this process, a bipolar-pulsed voltage is applied to the solution to create a non-equilibrium plasma field between the electrodes. This causes the synthesis of metal nanoparticles at the interface between the plasma and liquid phases. Using this method, we have successfully synthesized various nanoparticles, including Pt,^33–36^ PtPd,^21^ PdAu,^37^ Au,^34,38,39^ PtAg,^40^ and PtFe.^41^ The characteristic of this reaction process is that it utilizes a thermal non-equilibrium reaction. In other words, the advantage of SPP is that it can synthesize different non-equilibrium materials depending on the conditions of the reaction field, without being limited by thermal composition ratios or stability. From these points of view, the synthesis of spherical particles of PtPdIr ternary alloys with diameters of 1–3 nm using SPP can become an essential technology for the shape control of multicomponent alloy particles in the future.

In this study, we focused on the synthesis of PtPdIr ternary alloy nanoparticles using alloy electrodes in the solution plasma (SP) sputtering process, and attempted to control the size and composition ratio of spherical nanoparticles using SP sputtering process. To compare the characteristics of the obtained nanoparticles, we evaluated their catalytic performance for the ORR and HOR.

## 2. Experimental

### 2.1 Chemicals

Platinum, iridium, and palladium ICP standard solutions (1000 mg L^−1^), commercial Pt/C catalyst (20 wt%), and Nafion solution (5 wt% in a mixture of lower aliphatic alcohols and water) were purchased from Sigma-Aldrich, Germany. Sodium citrate dihydrate, acetone (99.5%), perchloric acid (HClO_4_, 70%), and isopropanol (IPA) were purchased from Kanto Chemical Co. Inc., Japan. PtIr (5 wt%, 10 wt%, 20 wt%) and PtPd (20 wt%) electrodes (diameter: 0.5 mm, 99.95% purity) were purchased from Nilaco Co., Japan.

### 2.2 SP process

**Fig. 1:**
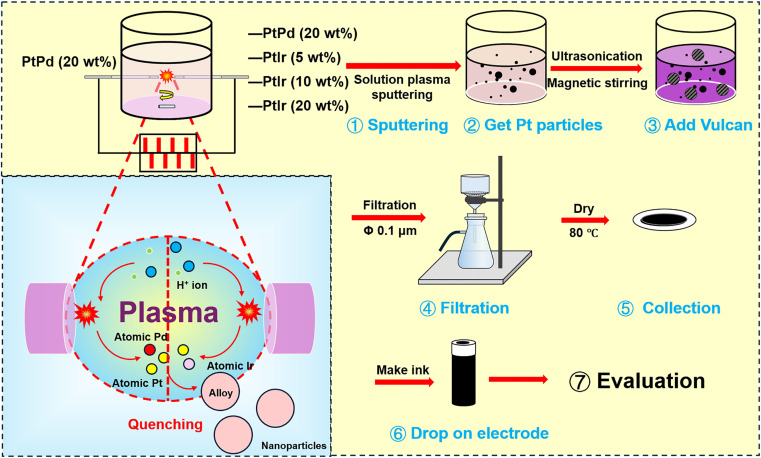
Experimental procedure for synthesis of PtPdIr nanoparticles.

The PtPd/C and PtPdIr/C alloy catalysts were synthesized by solution plasma sputtering as shown in Fig. 1. In the pin-to-pin discharge configuration, a PtPd wire served as one electrode, while PtPd, PtIr (5 wt%), PtIr (10 wt%), and PtIr (20 wt%) wires were used as the counter electrode. Both electrodes were wrapped in ceramic tubes to maintain a uniform discharge electric field, with a fixed distance of 1 mm between them. Aqueous solutions containing 1.5 mM sodium citrate dihydrate were used as stabilizers. The discharge was formed using a DC power supply (MPS-R06K01C-WP1-6CH, Kurita, Japan) with a repitation frequency of 30 kHz and a pulse width of 1.5 μs. After 20 minutes of discharge, the solutions containing PtPd and PtPdIr alloy particle were collected. Next, 20 mg of Vulcan was added to 80 mL of the solution containing the alloy, and the alloy particles were dispersed onto the Vulcan surface by ultrasonication and magnetic stirring. The solution and catalysts were then separated by filtration through a 0.1 μm membrane. Finally, the catalysts were dried in an oven at 80 °C.

### 2.3 Process and materials analysis

Optical emission spectroscopy (OES, UV/vis USB 2000+, Ocean Optics Inc., USA) and an oscilloscope (DLM2024, Yokogawa, Japan) were used to monitor the generation of free radicals during the plasma discharge and to record the current–voltage characteristics during the SP sputtering process. The mass fractions of Pt, Pd, and Ir in the nanparticles were determined by thermogravimetric analysis (TGA, C305759, SHIMADZU CORP., Japan) and inductively coupled plasma atomic emission spectroscopy (ICP-AES, SPS-7000; SEIKO, Japan). The morphology of nanoscale PtPd and PtPdIr particles was examined by high-resolution transmission electron microscopy (HR-TEM, JEM-2100F; JEOL Ltd., Japan). The phase composition was analyzed by X-ray diffraction (XRD, Rigaku Corp., Japan) using Cu Kα radiation (*λ* = 0.154 nm) at a scanning rate of 2° min^−1^ over a range of 10° to 90°. The electronic structure and elemental chemical bonding states were characterized by X-ray photoelectron spectroscopy (XPS, ESCALAB 250Xi; Thermo Fisher Scientific) using a Mg Kα X-ray source.

### 2.4 Electrochemical catalystic evaluation

To evaluate ORR and HOR performances of the synthesized nanoparticles, cyclic voltammetry (CV) and linear sweep voltammetry (LSV) were performed using a potentiostat with function generator (HAG1512m/BP, Hokuto Denko Co., Ltd., Japan). A 0.1 M HClO_4_ standard solution was used as the electrolyte for all electrochemical measurements. The catalysts tested in this study included PtPd and PtPdIr synthesized by SP sputtering and a commercial 20 wt% Pt/C catalyst used for comparison. The catalyst ink was prepared by mixing 3.5 mg catalyst, 0.75 mL isopropanol (IPA), 1.75 mL distilled water, and 20 μL Nafion (5 wt%). The mixture was ultrasonicated for one hour to ensure uniform dispersion. The ink was then dropped onto the surface of a glassy carbon rotating disk electrode (RDE, HAG1512m/BP, Hokuto Denko Co., Ltd., Japan) with a test area of 0.196 cm^2^ using a pipette. The working electrode was then dried on a spinner at 600 rpm. The catalyst loading was maintained at 20 μg cm^−2^ for all tests. During the electrochemical measurements, a platinum foil and a reversible hydrogen electrode (RHE) were used as counter and reference electrodes, respectively. Prior to the measurements, the electrolyte solution was flushed with N_2_ gas for 30 minutes to ensure saturation. The electrodes were then immersed in the solution for CV testing. CV scans were performed within a potential range of 0.05–1.2 V *vs.* RHE at a scan rate of 50 mV s^−1^. Following CV testing, O_2_ was bubbled into the electrolyte to saturation for subsequent CV and LSV measurements. LSV tests were performed at a speed of 1600 rpm with a potential range of 0.2–1.2 V *vs.* RHE., and a scan rate of 10 mV s^−1^.

## 3. Result and discussion

### 3.1 Crystal structure of the obtained nanoparticles

**Fig. 2:**
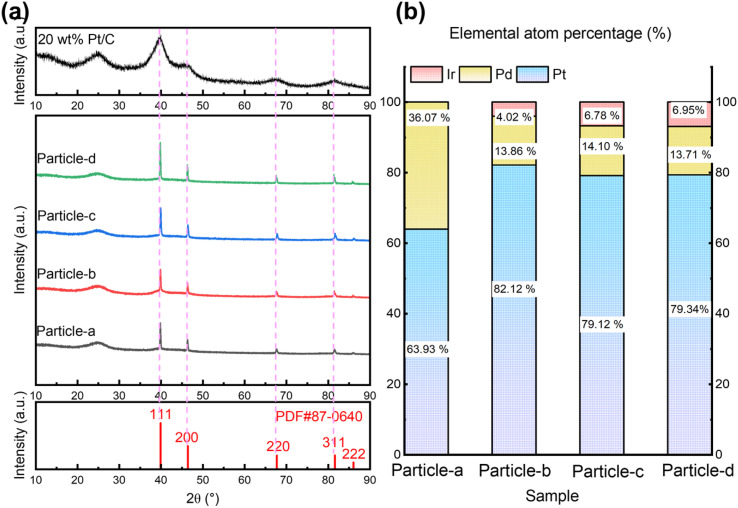
Crystal structure and the composition of nanoparticles obtained: (a) XRD patterns and (b) compositional atomic ratios by ICP-AES.

Fig. 2(a) and (b) show the XRD patterns and alloy composition ratios of the alloy particles synthesized by SP sputtering process, respectively. Particle-a, -b, -c, and -d in Fig. 2 are nanoparticles synthesized using PtPd, PtIr (5 wt%), PtIr (10 wt%), and PtIr (20 wt%) electrodes, respectively. In the XRD patterns in Fig. 2(a), the feature observed between 20° and 30° is a broad shoulder due to the carbon support. The almost identical diffraction patterns were observed with the following reflections at about 39.9°, 46.4°, 67.8°, and 81.6° in 2*θ*. These are due to the following *hkl* reflections: 111, 200, 220, and 311 (PDF#87-0640). These patterns represent a face-centered cubic (FCC) lattice. Furthermore, the similarity of these patterns to those of commercially available Pt/C (Pt content: 20 wt%) suggests that the metal particles produced by the SP sputtering procee are composed of a single metal phase. The Debye–Scherrer equation was used to estimate the average size of the Pt–Pd alloy nanoparticles according to the full half-peak width of 111 reflection.1
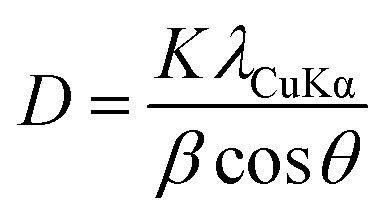
where *D* is the average crystal size of the nanometer, *K* is the shape factor (0.9 in this case), *λ* is the wavelength of the X-ray, *λ*_CuKα_ = 0.15406 nm, and *β* is the full width at half (FWHM) of the peak at 2*θ* in the pattern. The calculation results are listed in Table S1.† The atomic ratios of Pt, Pd and Ir in the samples are shown in Fig. 2(b). From the elemental ratios obtained by ICP-AES, it was found that when a PtIr alloy was used on one side of the electrode, Ir was incorporated into the produced nanoparticles. As the compositional ratio of Ir in the electrode increases, the compositional ratio of Ir in the nanoparticles also increases. However, around the time that the composition ratio of Ir in the electrode exceeds 10 wt%, the composition ratio of Ir in the nanoparticles becomes constant. In other words, despite the fact that the composition ratio of Ir in the sputtering electrode differs by a factor of two, the composition ratio of the nanoparticles in particle-c and particle-d is almost the same. The composition ratios of each nanoparticle are Pt : Pd : Ir in the order of particle-a (64 : 36 : 0), particle-b (82 : 14 : 4), particle-c (79 : 14 : 7) and particle-d (79 : 14 : 7). The numbers in brackets indicate the composition ratios of Pt : Pd : Ir.

### 3.2 Shape of obtained nanoparticles

**Fig. 3:**
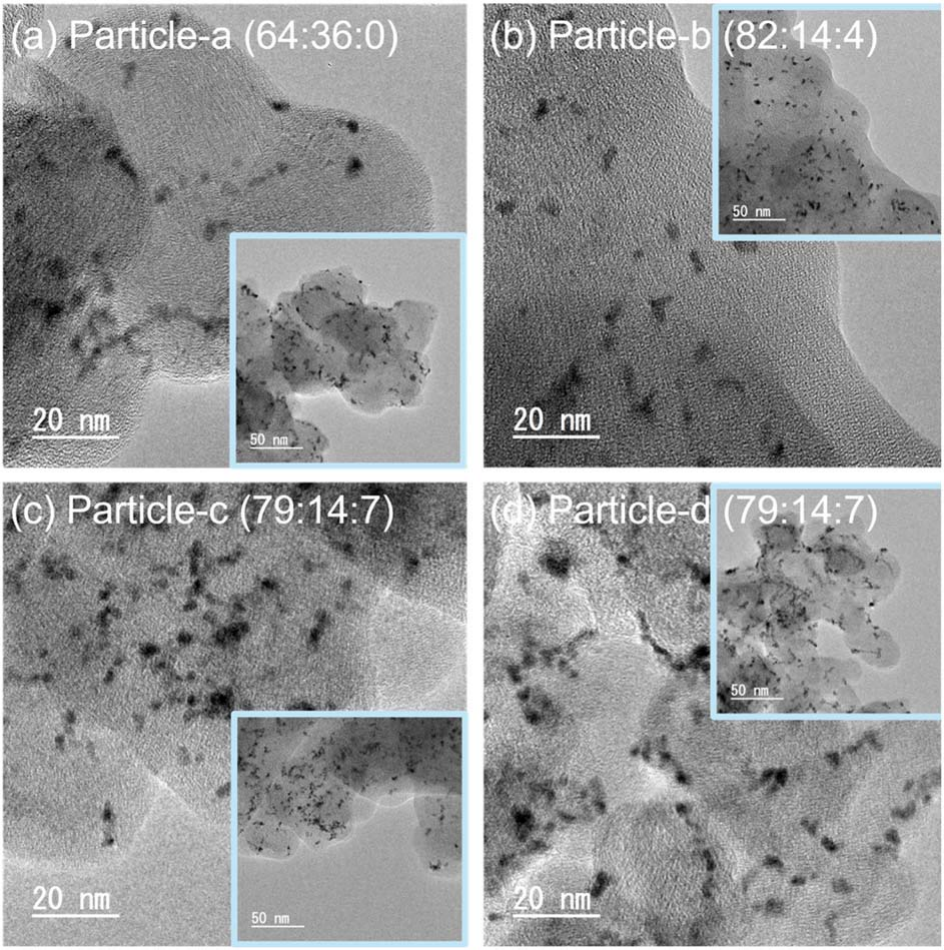
TEM images of (a) particle-a (64 : 36 : 0), (b) particle-b (82 : 14 : 4), (c) particle-c (79 : 14 : 7) and (d) particle-d (79 : 14 : 7). The inset images are the lower resolution TEM images.

The shapes of the nanoparticles synthesized by the SP sputtering process are shown in Fig. 3. Each image includes an enlarged view for more detailed observation of the shape of the nanoparticles and a low resolution TEM image. As shown in Fig. 3, most of the nanoparticles are spherical. However, some of the nanoparticles are slightly aggregated and spindle-shaped. The averaged particle size was about 1–3 nm. However, the particle size calculated by Scherrer method is 20–40 nm in Table S1.† The particle size determined by the Scherrer method represents the average size of crystallites (coherent crystalline regions) based on the diffraction peak width and thus does not necessarily match the actual physical dimensions of the particles. Conversely, the particle size obtained from TEM measurements is determined by directly observing the physically existing nanoparticles, thereby reflecting their true physical dimensions. The Scherrer method is generally applicable to crystallite sizes ranging from about 1 to 100 nm, with optimal accuracy within the range of approximately 3 to 80 nm. Particle sizes smaller than 1 nm typically result in excessively wide diffraction peaks, while particle sizes larger than 100 nm are heavily influenced by instrument resolution, making precise evaluation difficult. The observed difference between the particle sizes measured by TEM (approximately 3 nm) and those estimated by XRD (approximately 20 to 40 nm) is due to differences in measurement principles, the application range, and theoretical limitations inherent in the Scherrer method. Therefore, the discrepancy between particle sizes obtained *via* XRD and TEM measurements is considered reasonable. In practical evaluations of catalytic properties, particle size data obtained by TEM provides a more accurate representation of the actual physical particle size.

Fig. S1† shows the detail of the lattice space of Pt. Fig. S1(a-1)–(d-1)† are the original TEM image of particle-a (64 : 36 : 0), particle-b (82 : 14 : 4), particle-c (79 : 14 : 7), particle-d (79 : 14 : 7), respectively. Fig. S1(a-2)–(d1)† are the inverse Fourier transform (FFT) with the ring pattern of carbon on FFT was masked, and Fig. S1(a-3)–(d-3)† are the measurement results of the lattice distance. The diffraction fringes with lattice spacing distance of 0.21–0.22 nm.

### 3.3 Electrochemical catalytic evaluation of the obtained nanoparticles

#### 3.3.1 ORR performance

**Fig. 4:**
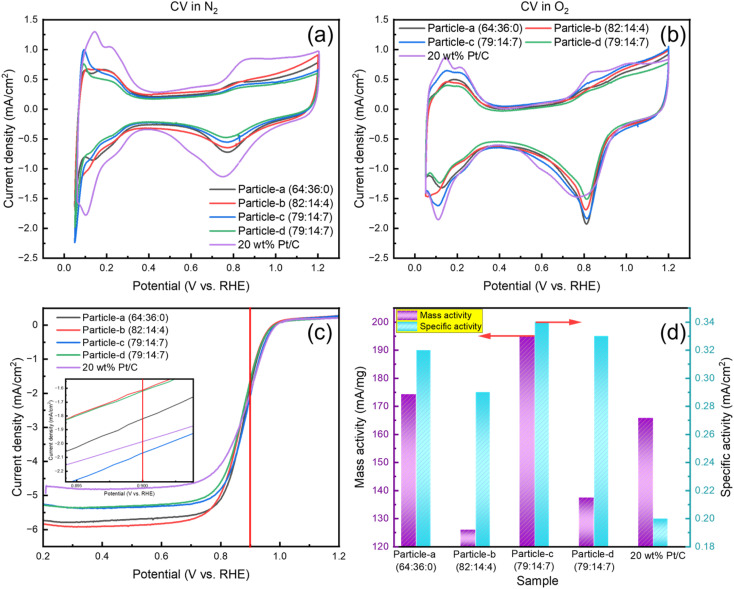
CV curves, LSV curves, and electrochemical activity for particle-a (64 : 36 : 0), particle-b (82 : 14 : 4), particle-c (79 : 14 : 7), particle-d (79 : 14 : 7), and 20 wt% Pt/C: (a) CV curve under N_2_ bubbling, (b) CV curve under O_2_ bubbling, (c) LSV curve under O_2_ bubbling with RDE with a speed of 1600 rpm and (d) mass and specific activities.

The catalytic ORR performance of the catalysts synthesized by SP sputtering process was evaluated by CV and LSV. Fig. 4(a) shows the CV curves recorded under N_2_ bubbling, and Fig. 4(b) shows the CV curves measured under O_2_ bubbling. Fig. 4(c) shows the LSV curves obtained under O_2_ bubbling at 1600 rpm. Fig. 4(d) compares the mass activity (MA) and specific activity (SA) of all samples. All measurements were performed with a constant metal loading of 20 μg cm^−2^. Table 1 summarizes all conditions and results of the ORR measurements. In Fig. 4(a) and (b), the potential range from 0 to 400 mV *vs.* RHE. corresponds to the hydrogen adsorption and desorption processes. The electrochemical active surface area (ECSA) can be obtained from the CV curves measured under N_2_ bubbling. The larger the hydrogen adsorption/desorption peak, the higher the ECSA, indicating that there are many active Pt surface sites available for catalytic reactions. The formula is as follows:^42–45^2
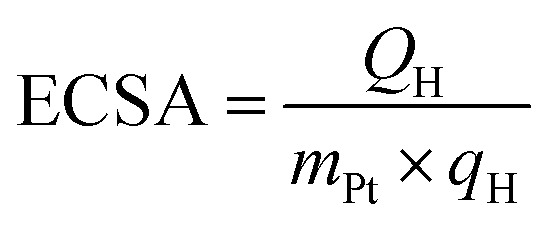
where *Q*_H_ is the coulombic charge for hydrogen desorption, *m*_Pt_ is the amount of Pt loaded, and *q*_H_ (210 μC cm^−2^) is the amount of charge required to oxidize a monolayer of hydrogen on the platinum site. Commercial 20 wt% Pt/C has the highest ECSA, which is about twice that of the sample synthesized using SP sputtering process. In the CV curves obtained in a saturated O_2_ environment, the ORR peaks are enhanced and a more pronounced enhancement is seen in particle-c (79 : 14 : 7) and particle-a (64 : 36 : 0). The peaks are shifted to the more positive potential position compared with the commercial 20 wt% Pt/C, indicating that the kinetics of ORR are faster, meaning that these catalysts promote ORR more efficiently. This indicates that these particles have excellent catalytic activity.

**Table 1:** Loading amount and oxygen reduction reaction performance results of all the catalysts^a^

Sample	Particle-a (64 : 36 : 0)	Particle-b (82 : 14 : 4)	Particle-c (79 : 14 : 7)	Particle-d (79 : 14 : 7)	20 wt% Pt/C
Pt : Pd : Ir (wt%)	76.2 : 23.8 : 0	87.7 : 8.1 : 4.2	84.3 : 8.6 : 7.1	84.6 : 8.0 : 7.4	100 : 0 : 0
Pt (wt%)	6.24	6.6	7.38	8.38	20
*m*_Pt_ (μg)	3.00	3.44	3.30	3.32	3.92
*E*_onset_ (V)	0.94	0.94	0.96	0.94	0.97
*E*_1/2_ (V)	0.88	0.87	0.88	0.87	0.89
MA(Pt) (mA mg^−1^)	174.32	126.04	195.34	137.51	165.80
ECSA (g m^−2^)	55.24	43.09	57.49	41.32	83.20
SA (mA cm^−2^)	0.32	0.29	0.34	0.33	0.20

a Pt wt%: Pt weight precent in the catalysts; *m*_Pt_: loading amount of Pt; *E*_onset_: onset potential; *E*_1/2_: half-wave potential; MA(Pt): mass activity of Pt; ECSA: electrochemical active surface area; SA: specific activity.

LSV is a method of quantitatively evaluating catalyst performance by measuring ORR activity under conditions where the rate of rotation is controlled. To minimize mass transfer restriction, the LSV curve was recorded at 1600 rpm. The inset in Fig. 4(c) is an enlarged LSV curve around 900 mV. A red vertical line has been drawn to indicate the reference potential for comparison. The half-wave potential (*E*_1/2_), which is the potential at which the current reaches half of its diffusion-limited current, is an important criterion for catalytic efficiency. Particle-c (79 : 14 : 7), particle-a (64 : 36 : 0) and 20 wt% Pt/C show more positive half-wave potentials. The MA at 900 mV can be determined using eqn (3) and (4):^46–48^3
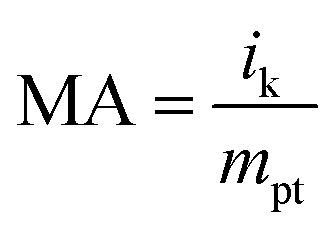
4
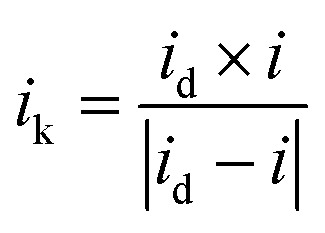
where *i*_k_ is the kinetic current and *m*_Pt_ is the load used in the measurement process. *i*_d_ is the diffusion limited current and *i* is the experimentally measured current at 0.9 V. PtPdIr nanoparticles are PtPd system with added Ir, and although it is assumed that they inherit the basic characteristics of PtPd system, in reality they have lower ORR activity than PtPd. In addition, particle-c (79 : 14 : 7) exhibits the highest MA(Pt) value, which has a high composition ratio of Ir. This value is higher than that of the original particle-a (64 : 36 : 0). However, the MA(Pt) value of particle-d (79 : 14 : 7) which was prepared using a PtIr (20 wt%) electrode, was significantly lower than that of particle-c (79 : 14 : 7). In terms of mass activity, the order was particle-c (79 : 14 : 7) > particle-a (64 : 36 : 0) > 20 wt% Pt/C > particle-d (79 : 14 : 7) > particle-b (82 : 14 : 4). A similar trend was observed in ECSA, but the variation in MA(Pt) was more pronounced. In other words, although the composition ratios are almost the same, differences in catalytic activity can be seen. This is thought to be due to the chemical bonding state of the surface, *etc.* Pt, Pd, and Ir are precious metals, and there are two possible methods of calculation for the mass activity value: MA(Pt + Pd + Ir) and MA(Pt). MA(Pt + Pd + Ir) of each sample is listed in Table S2.† In the case of this catalyst, Ir is included with the assumption that it can be used under harsh oxidation conditions such as HOR, ORR, and even OER, but even in this case, Pt remains the predominant active site responsible for catalytic activity, and the value of MA(Pt) often refers to the effective utilization rate, so it is often used. For this reason, MA(Pt) was used in this paper.

The two main parameters commonly used to characterize catalytic activity are MA and ECSA. MA refers to the current generated per unit mass of platinum in the oxygen reduction reaction, and ECSA refers to the active surface area per unit mass. However, both parameters can be significantly affected by the thickness of the catalyst film on the RDE during the test, which can lead to inaccuracies in the assessment of the intrinsic activity of the catalyst. To overcome this problem, specific activity, SA, is often used as a more reliable measure of intrinsic catalytic performance. This parameter reflects the catalytic current generated per unit area of catalyst surface. SA can be calculated from eqn (5):^49,50^5
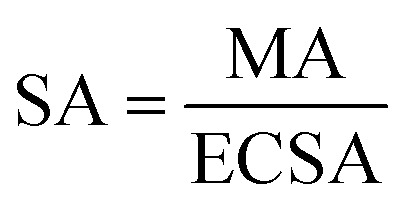


The particle-c (79 : 14 : 7) sample has the highest SA, which is 0.14 higher than that of the 20 wt% Pt/C. The particle-c (79 : 14 : 7) catalyst has the highest ORR catalytic activity. The order of activity is particle-c (79 : 14 : 7) > particle-d (79 : 14 : 7) > particle-a (64 : 36 : 0) > particle-b (82 : 14 : 4) > 20 wt% Pt/C.

#### 3.3.2 HOR performance

**Fig. 5:**
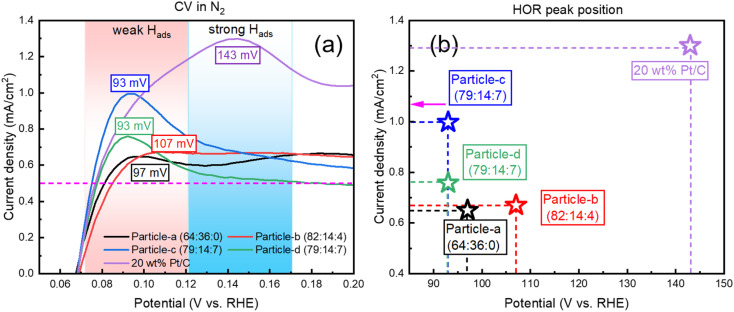
(a) Close-up CV curves and (b) peak position in HOR region for particle-a (64 : 36 : 0), particle-b (82 : 14 : 4), particle-c (79 : 14 : 7), particle-d (79 : 14 : 7), and 20 wt% Pt/C.

Next, PtPdIr (PtPd nanoparticles with added Ir) has low ORR activity in MA-based electrolytes. Fig. 5(a) shows the CV curve under N_2_ bubbling from 0.05 to 0.2 V. Since the standard electrode potential of HOR is 0 V *vs.* RHE, the closer the half-wave potential is to 0 V, the better the catalytic performance is. Furthermore, in particle-c (79 : 14 : 7), which has a higher composition ratio of Ir, the value of the *E*_1/2_ reaches its minimum value (0.074 mV in this study). This value is lower than that of the original particle-a (64 : 36 : 0) nanoparticles. However, the *E*_1/2_ value of the PtIr (20 wt%) electrode prepared particle-d (79 : 14 : 7) was slightly larger than that of particle-c (79 : 14 : 7). The 20 wt% Pt/C catalyst had the lowest performance (*E*_1/2_ = 0.08 mV), and the effect of adding Ir and Pd has a significant impact on improving the catalytic performance in the case of HOR. Fig. 5(b) shows the current density and potential corresponding to the HOR peak for different samples. The presence of Pd and Ir reduces the adsorption energy of hydrogen and shifts the oxidation peak to the negative direction. Compared with the 20 wt% Pt/C catalyst, the catalyst synthesized by the SP sputtering process has a lower oxidation current, but its reduction potential is close to 0 V *vs.* RHE. It is worth noting that particle-c (79 : 14 : 7) has the lowest *E*_onset_ and *E*_1/2_, indicating that it has excellent HOR catalytic performance. The order of HOR activity was as follows, the same as for ORR: particle-c (79 : 14 : 7) > particle-d (79 : 14 : 7) > particle-a (64 : 36 : 0) > particle-b (82 : 14 : 4) > 20 wt% Pt/C.

### 3.4 Chemical bonding states of the obtained nanoparticles

**Fig. 6:**
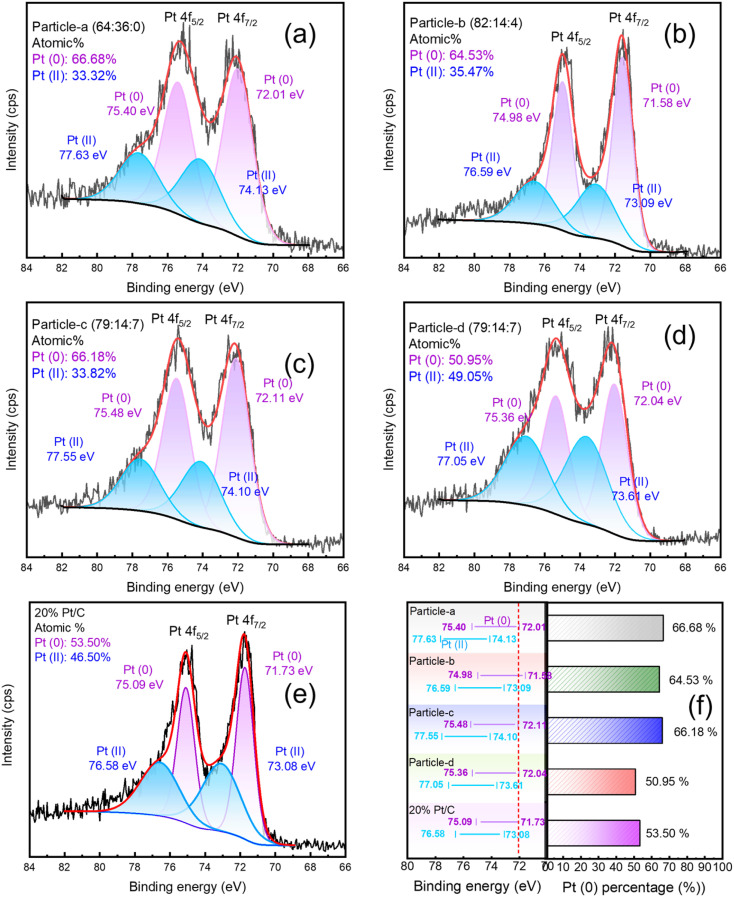
XPS spectra of Pt 4f (4f_5/2_ and 4f_7/2_) of (a) particle-a (64 : 36 : 0), (b) particle-b (82 : 14 : 4), (c) particle-c (79 : 14 : 7), (d) particle-d (79 : 14 : 7), (e) 20 wt% Pt/C, and (f) changes in chemical bonding states. In (a) to (e), the peaks obtained by fitting are also shown as Pt(0) and Pt(ii). In (f), the changes in chemical shifts and the ratios of Pt(0) are shown.

**Fig. 7:**
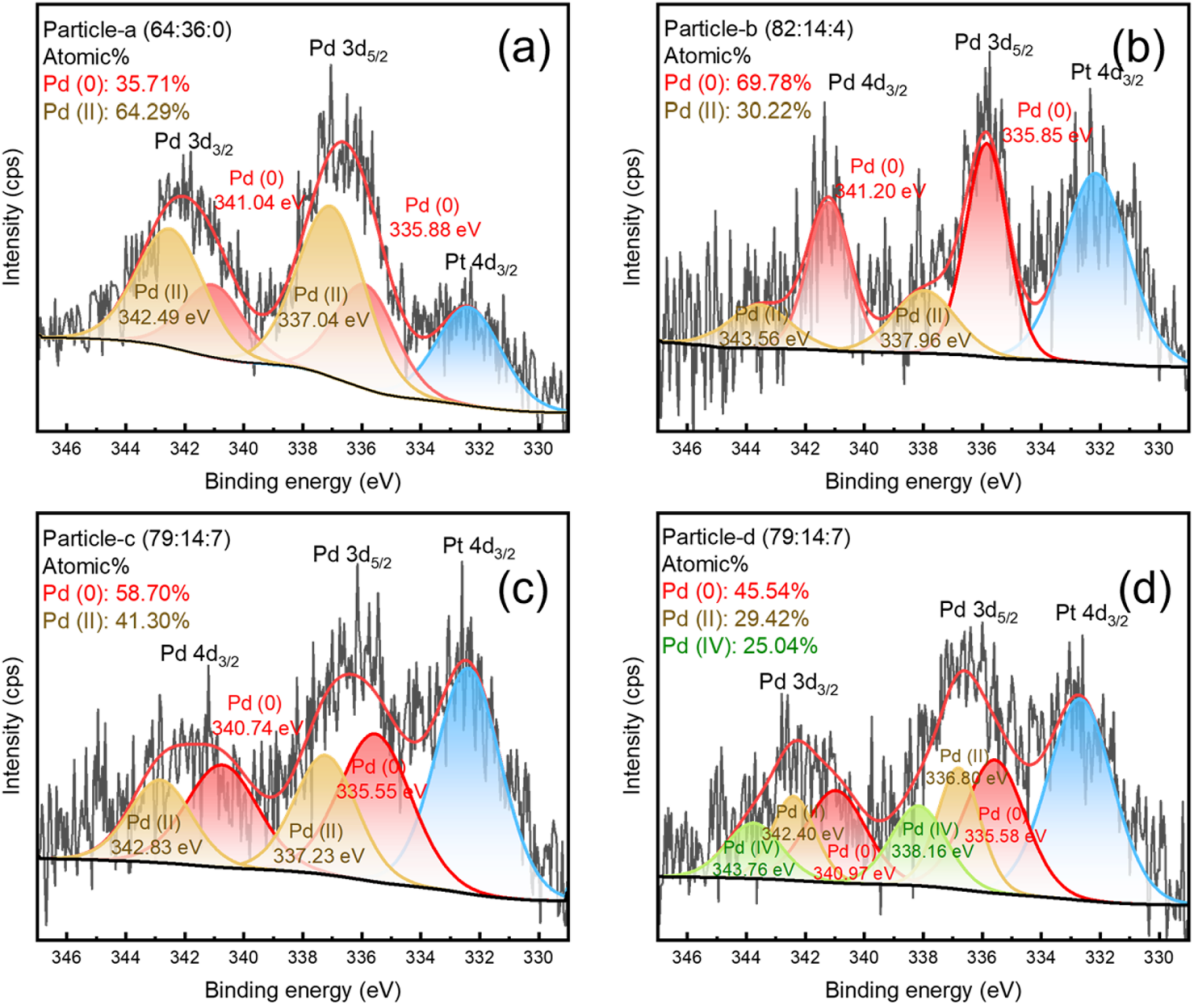
XPS spectra of Pd 3d (3d_5/2_ and 3d_3/2_) of (a) particle-a (64 : 36 : 0), (b) particle-b (82 : 14 : 4), (c) particle-c (79 : 14 : 7), (d) particle-d (79 : 14 : 7). The peaks obtained by fitting are also shown as Pd(0), Pd(ii) and Pd(iv).

**Fig. 8:**
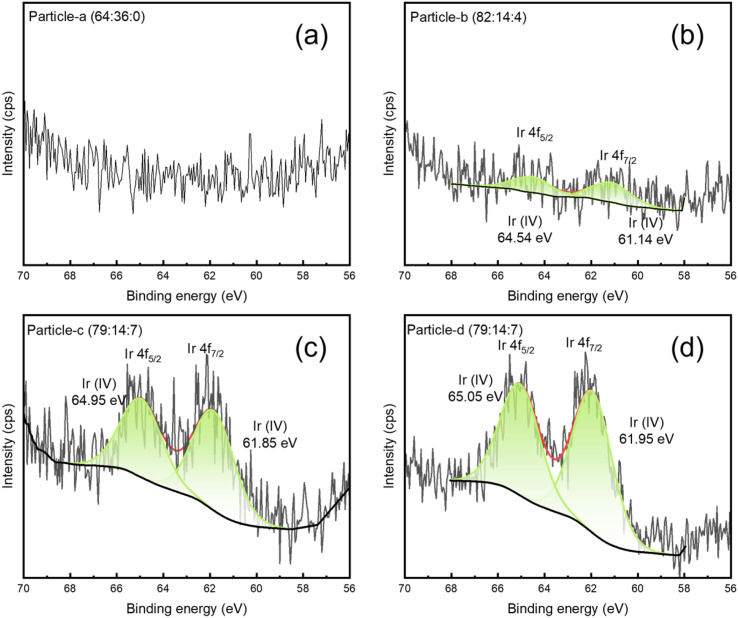
XPS spectra of Ir 4f (4f_5/2_ and 4f_7/2_) of (a) particle-a (64 : 36 : 0), (b) particle-b (82 : 14 : 4), (c) particle-c (79 : 14 : 7), (d) particle-d (79 : 14 : 7). The peaks obtained by fitting are also shown as Ir(iv).

Fig. 6(a)–(e), 7 and 8 show the XPS Pt 4f, Pd 3d and Ir 4f spectra of the alloy nanoparticles obtained with Vulcan and 20 wt% Pt/C, respectively. Fig. 6(f) shows the binding energies of Pt(0) and Pt(ii) for each sample on the left and the ratio of Pt(0) on the right. The Pt 4f_7/2_ and Pt 4f_5/2_ peaks can be resolved into Pt(0) and Pt(ii) components. The binding energies of Pt (0) and Pt(ii) in particle-a (64 : 36 : 0), c (79 : 14 : 7) and d (79 : 14 : 7) are shifted to higher values compared to 20 wt% Pt/C. This indicates that the chemical bonding states of Pt in these three samples are more diverse. In particular, particle-c (79 : 14 : 7) shows the highest binding energy, which is thought to be due to the significant change in electronic structure as a result of electrons transfer from Pd or Ir to Pt. In Fig. 7, the Pd 3d spectrum can be resolved into three valence states: Pd(0), Pd(ii), and Pd(iv). This is thought to be due to the high atomic ratio of Ir, which makes the chemical bonding state of Pd more complex. In Fig. 8, the Ir peak can be expressed as a single chemical bonding state of Ir(iv). This indicates that atomic oxygen (Ir–O) is adsorbed on Ir. Due to the low content of Ir, it cannot exist in a zero-valent state. In addition, as the amount of Ir increases, the peak intensity of the Ir-containing sample also increases. According to ICP-AES analysis, the composition ratio of particle-c (79 : 14 : 7) and particle-d (79 : 14 : 7) is almost the same, but XPS measurement shows that the Ir peak intensity of particle-d (79 : 14 : 7) is significantly stronger. This indicates that the composition ratio of iridium on the surface of particle-d (79 : 14 : 7) nanoparticles is high. In addition, the presence of the Pd(iv) peak indicates that Pd–O (adsorbed atomic oxygen) is formed on the Pd. From these observations, we can see that the main difference between particle-c (79 : 14 : 7) and particle-d (79 : 14 : 7) is the ratio of oxidized Pd that adsorbs O_2_ molecules to Pd(0) that does not. In particle-c (79 : 14 : 7), this ratio is balanced at 1 : 1. Furthermore, it was confirmed that the low Ir composition at the outermost surface of particle-c (79 : 14 : 7) is an important factor contributing to its excellent catalytic activity.

### 3.5 Relationship between plasma conditions and nanoparticles and their surfaces

**Fig. 9:**
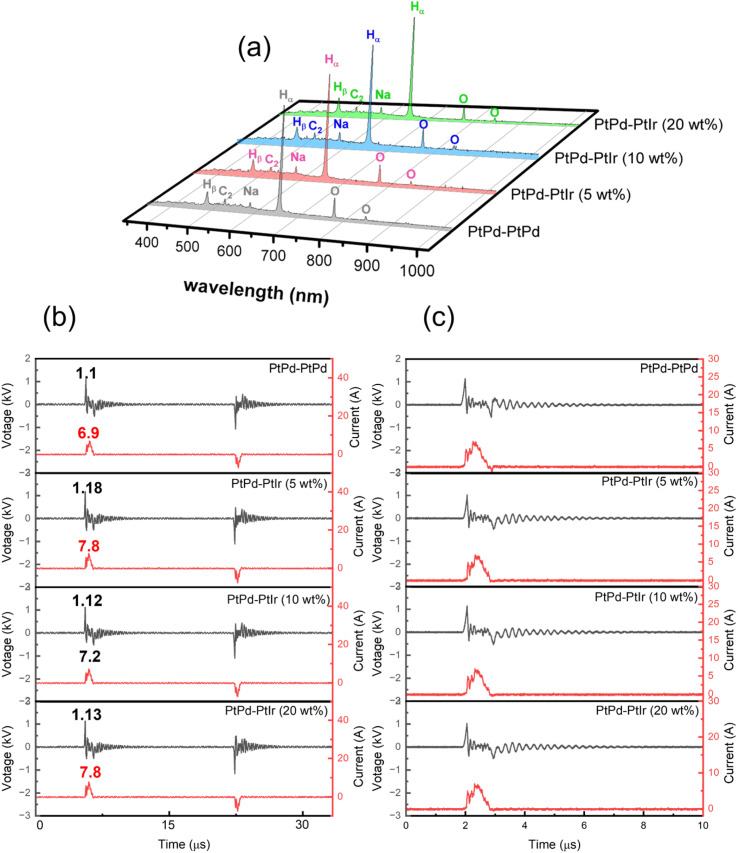
Plasma analysis during the synthesis: (a) the OES spectra for the solutions with different electrodes, (b) is *I*–*V* curves in one cycle and (c) is the *I*–*V* curves in a pulse.

As mentioned at the beginning, the principle of solution plasma sputtering is based on physical collision processes to produce nanoparticles, and the temperature of the ions and electrons in the plasma is particularly important. Ions collide with the electrode and atoms are ejected from the electrode surface. These excited atoms react with each other to form particles. The difference from the normal sputtering process is that it takes place at atmospheric pressure rather than in a vacuum, and because the plasma is surrounded by liquid at room temperature, it is a rapid cooling reaction. This rapid cooling reaction allows the size of the nanoparticles to be controlled within a few nanometers and also ensures high dispersibility. In other words, the state of the nanoparticles is determined by the state of the plasma. Therefore, the plasma temperature and the electron temperature in the solution plasma are indicated by OES, and the plasma state is studied in terms of the electron-ion balance (C_2_/H_α_) and (I_Na_). To consider the differences from a process perspective, the OES measurement results for each solution plasma condition are shown in Fig. 9. The C_2_, H_α_, and H_β_ emission lines could be measured, and the results were consistent with our previous research.^33^ From these results, the electron and gas temperatures were determined using the following equations.^51–53^6
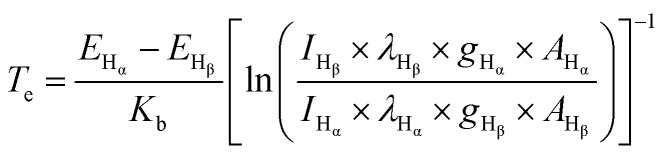
Here, *E* is the excitation energy, *K*_b_ is the Boltzmann constant (1.38 × 10^−23^ J K^−1^), *I* is the measured peak intensity, *λ* is the wavelength, and *A* is the transition probability of the active species.7
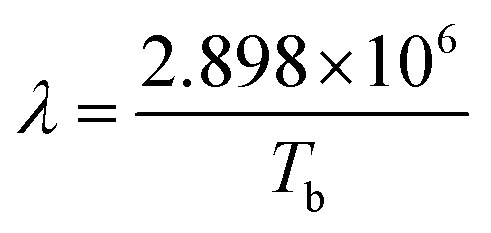


*λ* is the wavelength corresponding to the peak of the blackbody radiation. The actual current and voltage during the discharge process were recorded with an oscilloscope as shown in Fig. 9(b), and Fig. 9(c) shows the *I*–*V* curve for one pulse. The energy per pulse was calculated from the *I*–*V* curve, and the results of the OES analysis and the *I*–*V* curve calculation are summarized in Table 2. The energy obtained from the *I*–*V* curve in the case of the PtPd–PtPd electrode, the highest value was 432 (J per pulse). In the system with PtPd–PtIr arranged in the opposite direction, the energy decreases. On the other hand, the gas temperature also decreases and the electron temperature increases. To discuss the differences in the formation processes of particle-c (79 : 14 : 7) and particle-d (79 : 14 : 7), which will be discussed later, the C_2_/H_α_ ratio was highest and the Na radiation was strongest when the PtPd–PtIr (10 wt%) electrode pair was used. Therefore, it can be said that the system is closer to thermal equilibrium than when the PtPd–PtIr (20 wt%) electrode pair is used, but it is still in a non-equilibrium state. The degree of non-equilibrium in the plasma was the same for all systems except when the PtPd–PtIr (10 wt%) electrode pair was used. From these results, the difference in synthesis environment between particle-c (79 : 14 : 7) and particle-d (79 : 14 : 7) is that particle-d (79 : 14 : 7) was closer to thermal equilibrium. The sputtering yield (Ar^+^, 1 keV, normal incidence) was about 2.0 atoms per ion for Pt, about 2.2 to 2.5 atoms per ion for Pd, and about 1.7 to 1.8 atoms per ion for Ir, indicating that Ir is the least sputtered. This is due to the fact that Ir has the highest melting point. As mentioned above, the low Ir composition at the top surface of particle-c (79 : 14 : 7) is an important factor contributing to its excellent catalytic activity, and this can be explained by the fact that the energy difference in the synthesis of particle-c (79 : 14 : 7) and particle-d (79 : 14 : 7) changes the Ir concentration at the top surface. Furthermore, in order to understand why the plasma reaches thermal equilibrium when the proportion of Ir in the electrode is 10 wt%, it is essential to conduct further analysis of the interaction between Pt and Ir in the alloy electrode.

**Table 2:** Power per pulse, electron temperature, gas temperature, peak intensity of Na, and the peak-ratio of C_2_ to H_α_ intensities calculated based on the results in Fig. 9 a

Sample	Power per pulse (J)	*T*_e_ (K)	*T*_g_ (K)	C_2_/H_α_	I_Na_
Particle-a (64 : 36 : 0)	432.26	7800	5437	0.12	1054
Particle-b (82 : 14 : 4)	356.68	7840	5396	0.11	1318
Particle-c (79 : 14 : 7)	403.86	8889	5238	0.14	1617
Particle-d (79 : 14 : 7)	427.11	8951	4987	0.11	1122

a *T*_e_: electron temperature; *T*_g_: gas temperature.

From the point of view of durability, the stability of PtPdIr-based nanoparticles is extremely important for catalyst development. In this study, the PtPdIr/C system was found to be more stable than the Pt/C system, and even more stable than the PtPd/C system. Based on this premise, this study was conducted with a focus on process development. However, further investigation is needed to determine how the ability of controlling the diameter to 1–3 nm and the effect of plasma on the nanoparticle surface will affect durability.

Finally, in this study, we compared PtPd (64 : 36 : 0) and PtPdIr nanoparticles (82 : 14 : 4, 79 : 14 : 7, 79 : 14 : 7) with different Ir contents, and among these, particle c (79 : 14 : 7) showed the highest ORR and HOR activity. The following factors are thought to have contributed to this improvement in performance. These include: (1) a well-balanced electronic structure and d-band center, (2) the synergistic effect of each metal, (3) surface composition and adsorption sites, (4) particle size and dispersion, and (5) the plasma sputtering synthesis environment. Among them, the synergistic role of each metal on the surface was particularly important in points (2) and (3). Pt is the main active site for both ORR and HOR and shows high intrinsic activity. Pd tends to promote the adsorption and desorption of hydrogen (beneficial for HOR) and can also interact with oxygen species, so it complements the ORR activity of Pt. To achieve higher performance, the balance between Pd(ii), which can adsorb oxygen species, and Pd(0), which is metallic Pd, is thought to be important. Ir improves the overall stability and allows further fine-tuning of the electronic structure of Pt. Even a small amount of Ir(iv) on the surface, as confirmed by XPS, stabilizes the catalyst at more extreme potentials and contributes beneficially to the oxygen treatment step. The plasma sputtering synthesis environment was also important in determining the surface composition.

## 4. Conclusions

PtPdIr ternary alloy nanoparticles were synthesized by solution plasma sputtering. The synthesized particles were spherical with diameters of about 1–3 nm. By changing the electrode composition ratio during sputtering, the composition ratio of the nanoparticles could be controlled. The composition ratios were particle-a (64 : 36 : 0), particle-b (82 : 14 : 4), particle-c (79 : 14 : 7), and particle-d (79 : 14 : 7). When the load voltage conditions for plasma generation were kept constant, the ratio of Ir in the nanoparticles increased as the ratio of Ir in the electrode increased. However, the ratio stabilized at about 10 wt% Ir. The size of the nanoparticles could be controlled in the range of 1–3 nm. In addition, the nanoparticles were well dispersed when supported on carbon, and no agglomeration was observed. The electrochemical properties of the obtained nanoparticles in terms of ORR and HOR were investigated, and it was found that the particle-c (79 : 14 : 7) nanoparticles exhibited the highest ORR and HOR performance. In particular, the HOR properties were good. In the case of particle-a (64 : 36 : 0), the results of XPS measurements showed that the intensity of I_Pd(ii)_ > the intensity of I_Pd(0)_, and it was found that the ORR performance was supported by the adsorption of oxygen on Pd. On the other hand, in particle-b (82 : 14 : 4), the intensity of I_Pd(ii)_ ≪ the intensity of I_Pd(0)_, and in particle-c (79 : 14 : 7), the intensity of I_Pd(ii)_ = the intensity of I_Pd(0)_, so it can be concluded that the adsorption of oxygen on Pd affects the ORR performance and the HOR performance. Furthermore, when comparing particle-c (79 : 14 : 7) and particle-d (79 : 14 : 7), although the intensity of I_Pd(ii)_ = the intensity of I_Pd(0)_, the ratio of intensity of I_Pd(ii)_ decreases due to the formation of intensity of I_Pd(iv)_. The intensity of I_Pd(ii)_ correlated with the activity of ORR and HOR. The different ratio of intensity I_Pd(ii)_ and the different catalytic performance, despite the particles have the same elemental composition, suggest that the sputtering process changed the surface oxidation state ratio for some reason, such as ion and electron temperature. However, further detailed analysis is required.

## Data availability

The authors will supply the relevant data in response to reasonable requests.

## Author contributions

Yuanyuan Liu: conceptualization, methodology, data curation, writing original draft. Zhunda Zhu: methodology, formal analysis. Zhuoya Deng: methodology. Pengfei Wang: methodology. Sangwoo Chae: methodology. Yasuyuki Sawada: review & editing, funding acquisition. Nagahiro Saito: visualization, writing, review & editing, funding acquisition.

## Conflicts of interest

There are no conflicts to declare.

## Supplementary Material

RA-015-D5RA01747E-s001
