# Supplementary material for: Synthesis of size-controlled PtPdIr nanoparticles by solution plasma sputtering and their catalytic properties
Source: RSC Adv. 2025 Apr 22;15(16):12677–88. doi: 10.1039/d5ra01747e (PMC12012605; doi:10.1039/d5ra01747e)
Supplement: RA-015-D5RA01747E-s001 [file RA-015-D5RA01747E-s001.pdf]

Supplementary Information (SI) for RSC Advances.
This journal is © The Royal Society of Chemistry 2025

Supporting Information

Synthesis of size-controlled PtPdIr nanoparticles by solution plasma sputtering and their catalytic properties

Yuanyuan Liu,[a] Zhunda Zhu,[a] Zhuoya Deng,[a] Pengfei Wang,[a] Sangwoo Chae,[b] Yasuyuki Sawada,[a,b] and Nagahiro Saito[a,b,c,d*]

[a]Department of Chemical Systems Engineering, Graduate School of Engineering, Nagoya University, Furo-cho, Chikusa-ku, Nagoya 464-8603, Japan;

[b]Institute of Innovation for Future Society, Nagoya University, Furo-cho, Chikusa-ku, Nagoya 464-8603, Japan;

[c]Department of International Collaborative Program in Sustainable Materials and Technology for Industries between Nagoya University and Chulalongkorn University, Graduate School of Engineering, Nagoya University, Furo-cho, Chikusa-ku, Nagoya 464-8603, Japan;

[d]Conjoint Research Laboratory in Nagoya University, Shinshu University, Furo-cho, Chikusa-ku, Nagoya 464-8603, Japan.

*Corresponding Author: Nagahiro Saito: E-mail: saito.nagahiro.z7@f.mail.nagoya-u.ac.jp

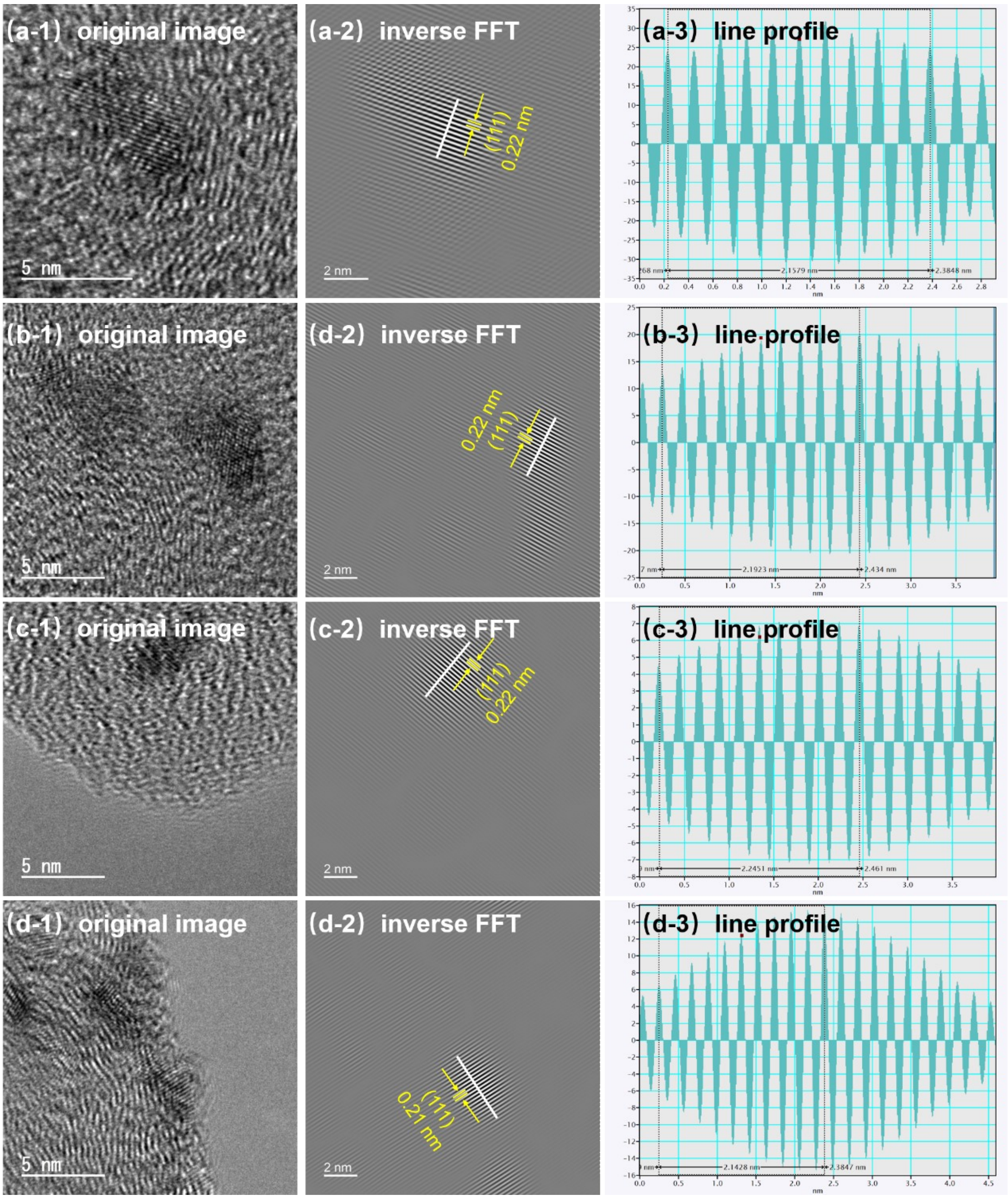

Fig. S1. Original TEM images of (a-1) particle-a (64:36:0), (b-1) particle-b (82:14:4), (c-1) particle-c (79:14:7) and (d-1) particle-d (79:14:7); (a-2)-(d-2) inverse Fourier Transform (FFT) masking the ring pattern of carbon on FFT, and (a-3)-(d-3) the measurement results of the lattice distance.

Table S1. Diffraction angles, half-maximum full width, and grain sizes obtained from 111 reflections in Fig. 2.

Sample	2θ (°)	FWHM (°)	Grain size (nm)
20 wt% Pt/C	39.70	1.166	7.24
particle-a (64:36:0)	39.83	0.234	36.2
particle-b (82:14:4)	39.83	0.406	20.8
particle-c (79:14:7)	39.91	0.257	32.9
particle-d (79:14:7)	39.80	0.212	39.9

FWHM: Full width at half of the peak at 2θ.

Table S2. Mass activity of Particles-a to -d based on Pt+Pd+Ir weight.

	Particle-a (64:36:0)	Particle-b (82:14:4)	Particle-c (79:14:7)	Particle-d (79:14:7)
MA(Pt+Pd+Ir) (mA/mg)	133.41	110.61	164.44	116.46

MA(Pt+Pd+Ir): Mass activity of Pt+Pd+Ir.
